# Receptor-binding autoantibodies are not associated with post-COVID fatigue

**DOI:** 10.1016/j.bbih.2026.101393

**Published:** 2026-10-23

**Authors:** Martin Achleitner, Juliane Dänhardt, Romina Kardashi, Nicole Toepfner, Katja de With, Zhenfei Li, Natalia Jarzebska, Roman N. Rodionov, Stefan R. Bornstein, Charlotte Steenblock

**Affiliations:** a Department of Internal Medicine III, University Hospital Carl Gustav Carus, Technische Universität Dresden, Dresden, Germany; b Department of Pediatrics, University Hospital Carl Gustav Carus, Technische Universität Dresden, Dresden, Germany; c Division of Infectious Diseases, University Hospital Carl Gustav Carus, Technische Universität Dresden, Dresden, Germany; d School of Cardiovascular and Metabolic Medicine and Sciences, Faculty of Life Sciences & Medicine, King's College London, London, UK; e Department of Endocrinology, Diabetology and Clinical Nutrition, University Hospital Zurich (USZ) and University of Zurich (UZH), Zurich, Switzerland

**Keywords:** COVID-19, Post-COVID-19 condition, Long COVID, Neurotransmitters, Autoantibodies, Fatigue

## Abstract

Persistent fatigue is a common symptom following SARS-CoV-2 infection, but its underlying mechanisms remain incompletely understood. Autoantibodies targeting G-protein coupled receptors and other neurotransmitter-related receptors have been proposed as potential biomarkers or mediators of post-COVID symptoms. However, their association with post-COVID fatigue remains unclear. Therefore, in the current study, we investigated receptor-binding autoantibodies against 13 neurotransmitter-related receptors in 206 participants with prior infection with SARS-CoV-2. Three groups of patients were enrolled: controls with no persistent fatigue, patients experiencing new-onset post-infection fatigue, and a group of patients who already suffered from fatigue before their infection with SARS-CoV-2. Fatigue was assessed using a standardized self-reported fatigue questionnaire. We found no significant differences in the concentrations of the measured receptor-binding autoantibodies between participants with new-onset fatigue, pre-existing fatigue, and controls without persistent fatigue. In multivariable analyses, none of the individual antibody concentrations was significantly associated with new-onset fatigue after adjustment for age, sex, and time since infection and correction for multiple testing. Individual antibody concentrations were also not associated with fatigue severity. The proportion of participants exceeding manufacturer-defined reference values did not differ significantly between the study groups. Similarly, cumulative antibody positivity was not associated with fatigue severity. In conclusion, in this cohort of SARS-CoV-2-infected participants, we found no evidence that concentrations of the measured receptor-binding autoantibodies are associated with post-COVID fatigue or fatigue severity. These findings do not support the use of these antibodies as stand-alone biomarkers of post-COVID fatigue. Because all participants had previously experienced SARS-CoV-2 infection, the study cannot determine whether infection itself alters antibody concentrations. Furthermore, the binding assays do not establish whether the detected antibodies have functional receptor activity. These findings underscore the urgent need for longitudinal studies with comprehensive clinical phenotyping and functional antibody assays are needed to further investigate the potential role of receptor-binding autoantibodies in post-COVID symptoms.

## 1 Introduction

Persistent post-COVID-19 condition, commonly known as long COVID, poses a dual challenge, both medical and socioeconomic, for healthcare systems across the globe. Estimates of long COVID prevalence vary significantly, ranging from a few percent to approximately 50% of all individuals infected with SARS-CoV-2 (Bornstein et al., 2022). These numbers may differ depending on study methodologies, population characteristics, and how the condition is defined. According to the World Health Organization, 2022 (WHO), 10–20% of individuals experience at least one symptom persisting or emerging more than three months after a SARS-CoV-2 infection (WHO, 2022). The latest report from the Centers for Disease Control and Prevention (CDC) based on data from the U.S. Census Bureau's Household Pulse Survey, as of September 2024, states that approximately 17% of patients have reported experiencing long COVID after infection with SARS-CoV-2 (CDC, 2024). Long COVID often manifests with symptoms such as fatigue and muscle weakness, which are reminiscent of those observed in post-infectious myalgic encephalomyelitis/chronic fatigue syndrome (ME/CFS) following infectious diseases caused by a number of viruses, such as Epstein-Barr (EBV), Ebola, Dengue, polio, SARS-CoV-1, Chikungunya, West Nile, Ross River, and enteroviruses (Choutka et al., 2022). Also, non-viral pathogens such as *Coxiella burnetti*, *Borrelia* and *Giardia lamblia* are known to trigger post-acute infection syndrome (PAIS) (Choutka et al., 2022).

A recent multicenter study has shown no evidence that the proportion of individuals with ME/CFS-like illness differs between those infected with SARS-CoV-2 and those without SARS-CoV-2 infection up to 12 months post-infection (Unger et al., 2024). Nevertheless, due to the similarity of symptoms present in ME/CFS and long COVID, a common etiopathogenesis has been suggested (Poenaru et al., 2021; Steenblock et al., 2022). The exact pathology of ME/CFS remains unknown, but it is believed to result from the dysregulation of multiple systems triggered by a specific event (Arron et al., 2024). The initiation of disease in ME/CFS through infection, persistent immune activation, and dysregulation of the autonomic system suggests an autoimmune condition targeting neurotransmitter receptors (Loebel et al., 2016; Wilhelm et al., 2026).

Researchers are struggling to find effective therapies against both ME/CFS and long COVID, which is further complicated because of the complexity of symptoms and lack of specific biomarkers (Scheibenbogen et al., 2023). In pathogenesis, the induction of autoantibodies against G-protein coupled receptors (GPCR), such as β-adrenergic receptors (β-adr-R), has been suspected, as such autoantibodies were shown to play a pathogenic role in several autoimmune diseases (Loebel et al., 2016). Previous studies have shown that the concentration of autoantibodies against neurotransmitter receptors can be decreased using extracorporeal apheresis (Achleitner et al., 2023; Korth et al., 2024; Steenblock et al., 2025; Stein et al., 2025), and that this appears to have a beneficial effect in a subset of patients with ME/CFS (Achleitner et al., 2023; Stein et al., 2025), further implicating that these autoantibodies might be at least partly involved in the underlying mechanisms contributing to fatigue.

In the current study, we expanded our previous investigations (Achleitner et al., 2023; Korth et al., 2024) and tested whether autoantibodies against 13 different neurotransmitter-related receptors are associated with the presence of persistent fatigue after infection with SARS-CoV-2. Importantly, our study specifically addresses fatigue as a symptom and was not designed to diagnose ME/CFS; participants were therefore not characterized according to formal ME/CFS diagnostic criteria.

## 2 Methods

### 2.1 Study cohort and fatigue assessment

The total sample size for the study was N = 206 participants, with n = 144 adult patients from the long COVID outpatient clinic at the University Hospital in Dresden, Germany, that reported newly developed fatigue. These patients tested positive for SARS-CoV-2 by PCR and reported experiencing fatigue symptoms for at least 4 weeks after the initial infection. Furthermore, n = 24 patients with pre-existing fatigue before infection with SARS-CoV-2 were included. A control group consisted of n = 38 participants (employees of the University Hospital in Dresden), who had also experienced a confirmed infection with SARS-CoV-2 but developed only mild or asymptomatic COVID-19 and reported no symptoms of fatigue. No formal sample size or power calculation was performed for the control group. The number of controls was determined by the number of eligible volunteers available during the study period. The control group was recruited independently of the long-COVID outpatient cohort.

Fatigue in all patients was assessed by self-report. A standardized questionnaire FACIT-F (https://www.facit.org/measures/FACIT-F) approved for the assessment of fatigue in autoimmune diseases (Chauffier et al., 2012) was used to measure the degree of fatigue. The analysis focused exclusively on the fatigue-related items of the questionnaire, which comprised six questions, with a total score ranging from 0 to 24 (each item was scored from 0 to 4). Higher scores indicating greater fatigue. No transformation of the score was performed. No predefined threshold for clinically meaningful fatigue was used; the FACIT-F score was used as a continuous measure of self-reported fatigue severity and was not used to establish a diagnosis of ME/CFS. The DePaul Symptom Questionnaire (DSQ) and the Multidimensional Fatigue Inventory were not administered. Consequently, other clinical features relevant to ME/CFS, including post-exertional malaise, unrefreshing sleep, cognitive impairment, orthostatic intolerance, and functional impairment, were not systematically assessed and could not be evaluated as part of this study.

All participants provided written informed consent and all methods were performed in accordance with the relevant guidelines and regulations. Research was performed in accordance with the Declaration of Helsinki. The study was approved by the TU Dresden ethical committee (BO-EK-193052024).

### 2.2 Neurotransmitter receptor-binding autoantibody measurements in patient plasmas

Blood was collected from all patients. Antibodies against α1, α2a, α2b, α2c, β1, and β2 adr-Rs, angiotensin II type 1 receptor (AT1R), endothelin receptor A (ETAR), and the muscarinic acetylcholine receptors (AChRs) 1, 2, 3, 4, and 5 were measured in the plasma with commercially available immunoassays (CellTrend GmbH, Luckenwalde, Germany) according to the instructions of the manufacturer.

### 2.3 Statistical analysis

Continuous variables were summarized as median and interquartile range (IQR), and categorical variables as counts and percentages. Differences in continuous variables across the three study groups (control, pre-existing fatigue, and new-onset fatigue) were assessed using the Kruskal–Wallis test, followed by Dunn's multiple-comparison test with Holm adjustment where appropriate. Sex distributions were compared using Fisher's exact test. For comparisons of continuous variables between two independent groups, the Mann–Whitney *U* test was used.

Antibody levels were compared between the three groups using the Kruskal–Wallis test, with adjustment for multiple comparisons using the Benjamini–Hochberg false discovery rate (FDR) procedure across the 13 antibody measurements.

To assess the association between antibody levels and new-onset fatigue, binary logistic regression models were fitted with new-onset fatigue as the outcome. Antibody concentrations were log2-transformed such that the resulting odds ratios (ORs) represent the change in odds of new-onset fatigue associated with a two-fold increase in antibody level. Each antibody was analyzed in a separate multivariable logistic regression model adjusted for age, sex, and time since SARS-CoV-2 infection. The control and pre-existing fatigue groups were coded as the reference outcome (0), whereas the new-onset fatigue group was coded as the outcome (1).

The assumption of a linear association between continuous predictors and the log odds of new-onset fatigue was assessed by comparing models containing linear terms with models incorporating natural cubic spline terms using likelihood-ratio tests. Age did not show evidence of nonlinearity and was therefore retained as a linear term. In contrast, time since SARS-CoV-2 infection showed evidence of a nonlinear association and was modeled using a natural cubic spline with three degrees of freedom. The final models therefore included antibody level (log2-transformed), age, sex, and a natural cubic spline for time since infection.

Odds ratios (ORs) were calculated by exponentiating the regression coefficients, and 95% confidence intervals (CIs) were obtained from the fitted models. *P* values were adjusted for multiple testing across the 13 antibodies using the Benjamini–Hochberg FDR procedure.

To investigate a potential cumulative association between autoantibody positivity and fatigue severity, an exploratory cumulative autoantibody positivity score was calculated for each participant. For the primary cumulative analysis, eight autoantibodies available for the majority of participants were considered (AT1R, ETAR, α1-adr-R, α2a-adr-R, α2b-adr-R, α2c-adr-R, β1-adr-R, and β2-adr-R). For each antibody, positivity was defined as a concentration exceeding the manufacturer-defined reference value. The cumulative positivity score corresponded to the number of these eight autoantibodies exceeding their respective reference values.

Participants were additionally categorized according to cumulative positivity as having 0, 1, 2, or ≥3 autoantibodies above the respective reference values. Fatigue scores were compared between these categories using the Kruskal–Wallis test. To account for potential confounding by age, sex, and time since infection, multivariable linear regression was performed with fatigue score as the dependent variable and cumulative autoantibody positivity, age, sex, and time since infection as independent variables. Regression coefficients (β), 95% CIs, and *P* values were calculated. This analysis was considered exploratory.

All statistical analyses were performed in R version 4.6.1 and in GraphPad Prism version 10.2.3.

## 3 Results

### 3.1 Patient characteristics

To investigate whether patients with post-COVID fatigue have increased levels of autoantibodies against neurotransmitter receptors, we measured autoantibody concentrations against 13 different neurotransmitter receptors in the plasma of 144 patients experiencing new-onset fatigue for at least 4 weeks after COVID-19, 24 patients, who had already experienced fatigue before their infection with SARS-CoV-2, and in 38 control patients who were infected with SARS-CoV-2 but did not experience persistent fatigue (Table 1). Median age was 36.6 years (IQR, 31.0–42.3) in controls, 47.6 years (33.1–53.1) in participants with pre-existing fatigue, and 46.5 years (37.0–54.1) in those with new-onset fatigue (Kruskal–Wallis *P* < 0.001). Dunn's post-hoc tests with Holm correction showed that both the pre-existing fatigue group (*P* = 0.011) and the new-onset fatigue group (*P* < 0.001) were older than controls, whereas age did not differ between the two fatigue groups (*P* = 0.736) (Fig. 1A, Table 1). Women were the majority in all three groups, but there were no statistically significant differences in sex distribution between the groups (Table 1, Fisher's exact test, *P* = 0.7369). When comparing males and females within the three groups, the mean age of females in the new-onset fatigue group was significantly lower than that of males (Fig. S1A, Mann-Whitney *P* = 0.0093), whereas in the control and pre-existing fatigue groups, no statistically significant age differences between males and females were observed.

**Table 1:** Patient data.

Characteristic	Control (n = 38)	Pre-existing fatigue (n = 24)	New-onset fatigue (n = 144)	*P* value
**Age, years**	36.56 (30.98–42.31)	47.55 (33.14–53.11)	46.54 (37.03–54.13)	<0.001
**Female sex, n (%)**	30 (78.9%)	18 (75.0%)	116 (80.6%)	0.7369
**Time since positive SARS-CoV-2 test, days**	112.5 (90–133.75)	234.5 (142.75–359.25)	358 (206–521.5)	<0.001
**Assessed ≥90 days after SARS-CoV-2 infection, n (%)**	29 (76.3%)	22 (91.7%)	139 (96.5%)	<0.001
**Fatigue score**	2 (1–3)	15.5 (12.75–18)	16 (12.75–19)	<0.001

Values are presented as median (IQR), where IQR denotes interquartile range. *P* values were calculated using the Kruskal–Wallis test for continuous variables and Fisher's exact test for categorical variables.

**Fig. 1:**
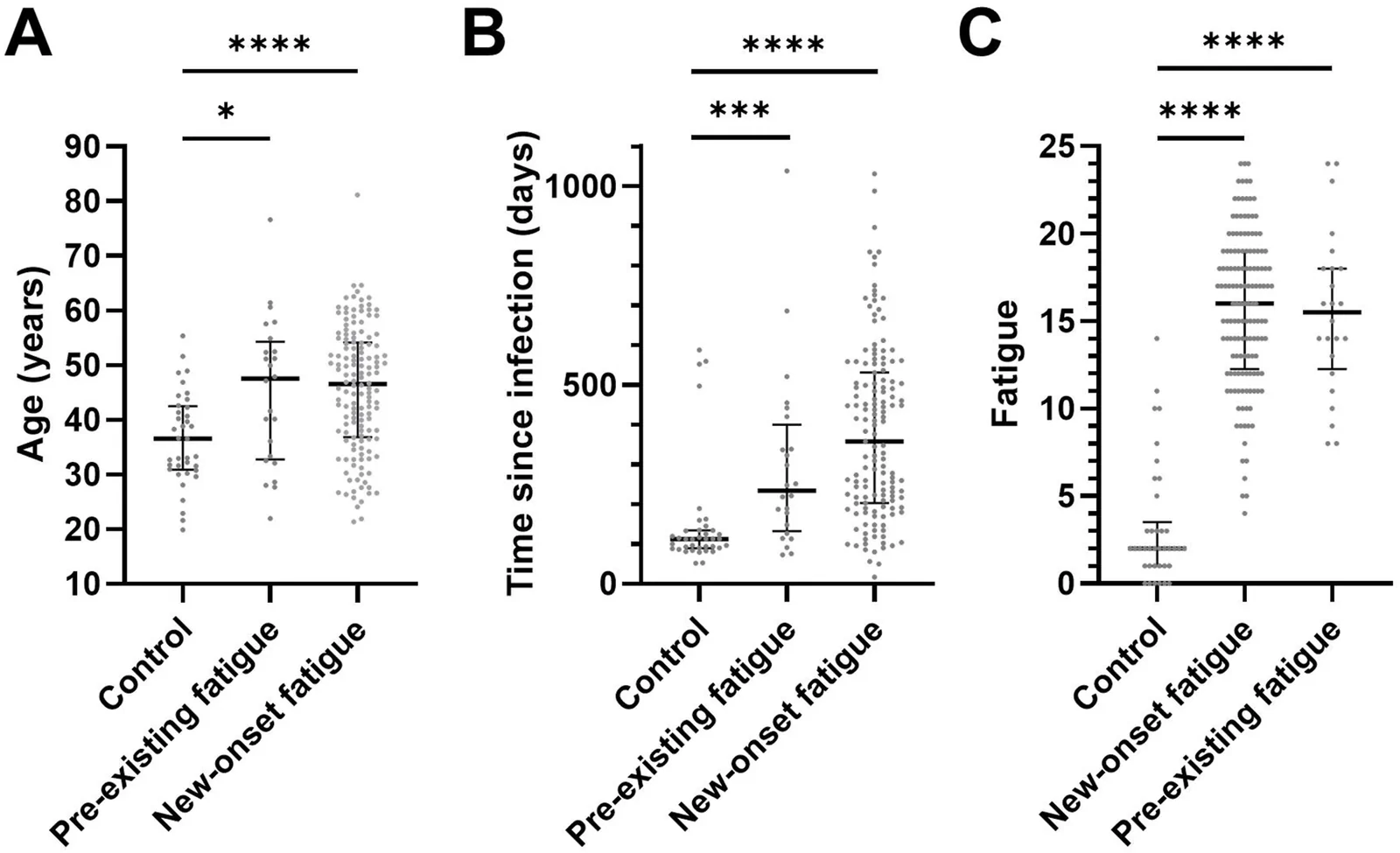
**Age, time since SARS-CoV-2 infection, and fatigue severity in the study groups.** A) Age distribution in control participants, participants with pre-existing fatigue, and participants with new-onset fatigue. B) Time since SARS-CoV-2 infection in the three study groups. C) Fatigue scores in the three study groups. Individual data points represent individual participants; horizontal lines indicate the median, and error bars indicate the interquartile range. Group comparisons were performed using the Kruskal–Wallis test followed by Dunn's multiple-comparison test with Holm adjustment. *P < 0.05; ***P < 0.001; ****P < 0.0001.

Median time since infection was 112.5 (90–133.75) days in controls, 234.5 (142.75–359.25) days in participants with pre-existing fatigue, and 358 (206–521.5) days in participants with new-onset fatigue (Kruskal–Wallis *P* < 0.001). In post-hoc Dunn's tests with Holm adjustment, time since infection was significantly longer in the pre-existing fatigue group compared with controls (*P* = 0.0045) and in the new-onset fatigue group compared with controls (*P* < 0.001), as well as in the new-onset fatigue group compared with the pre-existing fatigue group (*P* = 0.0475) (Fig. 1B–Table 1). When comparing males and females, no statistically significant differences in the time since infection were observed (Fig. S1B). At the time of assessment, the proportion of participants assessed ≥90 days after SARS-CoV-2 infection differed significantly between groups (76.3% of controls, 91.7% of participants with pre-existing fatigue, and 96.5% of participants with new-onset fatigue; Fisher's exact test, *P* < 0.001). Still, the majority of participants were assessed at least 3 months after SARS-CoV-2 infection, corresponding to the temporal component of the WHO clinical case definition for post-COVID-19 condition (Table 1).

As expected, fatigue scores were substantially higher in both fatigue groups than in controls (median 15.5 and 16.0 vs. 2.0; Kruskal–Wallis *P* < 0.001) (Fig. 1C–Table 1). In all groups, there were no statistically significant differences in the reported fatigue scores between males and females (Fig. S1C).

These results show that the control group is not perfectly matched to the two fatigue groups in terms of age and time since the initial SARS-CoV-2 infection and that there is a sex difference in relation to age. Therefore, age, sex, and time since infection were included as covariates in all multivariable analyses.

### 3.2 No significant differences in neurotransmitter receptor autoantibody concentrations between fatigue groups

This study aimed to explore a potential association between fatigue symptoms following SARS-CoV-2 infection and concentrations of antibodies targeting various neurotransmitter receptors. We measured the levels of 13 different autoantibodies (α1, α2a, α2b, α2c, β1, and β2 adr-Rs, M1, M2, M3, M4, and M5 AChRs, AT1R, and ETAR) in the plasma of control, new-onset fatigue and pre-existing fatigue subgroups. Of note, as the kits for measuring M1, M2, and M5 AChRs were developed later, these autoantibodies were not measured in all patients. Therefore, M1R-Ab, M2R-Ab, and M5R-Ab analyses were based on substantially smaller subsets of participants (n = 53 overall). No significant differences in antibody levels were observed between the three groups for any of the 13 autoantibodies examined (all Kruskal–Wallis *P* ≥ 0.265; all FDR-adjusted *P* = 0.933; Table 2). The largest numerical difference was observed for α2b-adr-R, which was higher in the new-onset fatigue group, but this difference was not statistically significant (*P* = 0.265; FDR-adjusted *P* = 0.933).

**Table 2:** Autoantibody concentrations.

Autoantibody	Control	Pre-existing fatigue	New-onset fatigue	P value	FDR-adjusted P
AT1R	11.25 (8.70–16.91), n = 38	9.10 (8.12–13.49), n = 22	11.36 (8.36–15.69), n = 144	0.836	0.933
ETAR	10.59 (8.47–13.83), n = 38	10.03 (7.62–12.31), n = 22	10.52 (8.12–14.05), n = 144	0.880	0.933
α1-adr-R	9.27 (7.43–14.77), n = 38	8.44 (6.51–21.64), n = 22	9.47 (6.71–17.39), n = 144	0.933	0.933
α2a-adr-R	9.38 (7.03–11.55), n = 38	8.26 (7.11–10.41), n = 22	8.79 (6.42–11.95), n = 144	0.828	0.933
α2b-adr-R	7.63 (5.74–10.62), n = 38	7.68 (5.53–13.12), n = 22	9.08 (6.50–13.02), n = 144	0.265	0.933
α2c-adr-R	6.08 (4.68–8.37), n = 38	5.62 (3.91–8.55), n = 22	5.92 (4.39–9.23), n = 144	0.896	0.933
β1-adr-R	9.17 (5.90–12.94), n = 38	8.11 (5.38–12.05), n = 24	8.99 (5.94–14.38), n = 144	0.912	0.933
β2-adr-R	16.88 (10.35–24.85), n = 38	12.61 (8.28–19.66), n = 24	13.67 (8.43–27.30), n = 144	0.610	0.933
M1R	7.29 (6.66–7.44), n = 3	8.43 (5.79–9.99), n = 7	6.14 (4.49–9.03), n = 43	0.802	0.933
M2R	7.27 (6.78–8.37), n = 3	7.13 (5.30–7.79), n = 7	6.87 (5.05–9.18), n = 43	0.815	0.933
M3R	11.86 (8.95–20.21), n = 38	9.50 (7.43–15.49), n = 24	11.01 (7.40–18.47), n = 144	0.463	0.933
M4R	9.03 (7.03–11.56), n = 38	8.62 (6.27–14.92), n = 24	9.65 (7.24–12.93), n = 144	0.760	0.933
M5R	9.68 (9.04–12.50), n = 3	10.32 (9.91–12.92), n = 7	10.40 (8.18–13.12), n = 43	0.892	0.933

Values are median (IQR). *P* values were calculated using the Kruskal–Wallis test. *P* values were adjusted for multiple comparisons using the Benjamini–Hochberg false discovery rate (FDR) procedure.

### 3.3 Autoantibody concentrations are not associated with new-onset fatigue after adjustment for covariates

To determine whether autoantibody levels were independently associated with new-onset fatigue, we performed multivariable logistic regression analyses adjusted for age, sex, and time since SARS-CoV-2 infection. Because the association between time since infection and new-onset fatigue was non-linear, time was modeled using a natural spline with three degrees of freedom. Autoantibody concentrations were log2-transformed, and effect estimates therefore represent the change in odds of new-onset fatigue associated with a two-fold increase in antibody concentration.

None of the 13 autoantibodies was significantly associated with new-onset fatigue after adjustment for covariates and correction for multiple testing (all FDR-adjusted *P* ≥ 0.718). For AT1R, a two-fold increase in antibody concentration was associated with an OR of 1.14 (95% CI 0.72–1.81; *P* = 0.572; FDR-adjusted *P* = 0.718). Similarly, estimates for the remaining autoantibodies ranged from OR 0.34 to 1.25, with all 95% CIs crossing 1 and all FDR-adjusted *P* values ≥ 0.718 (Fig. 2).

**Fig. 2:**
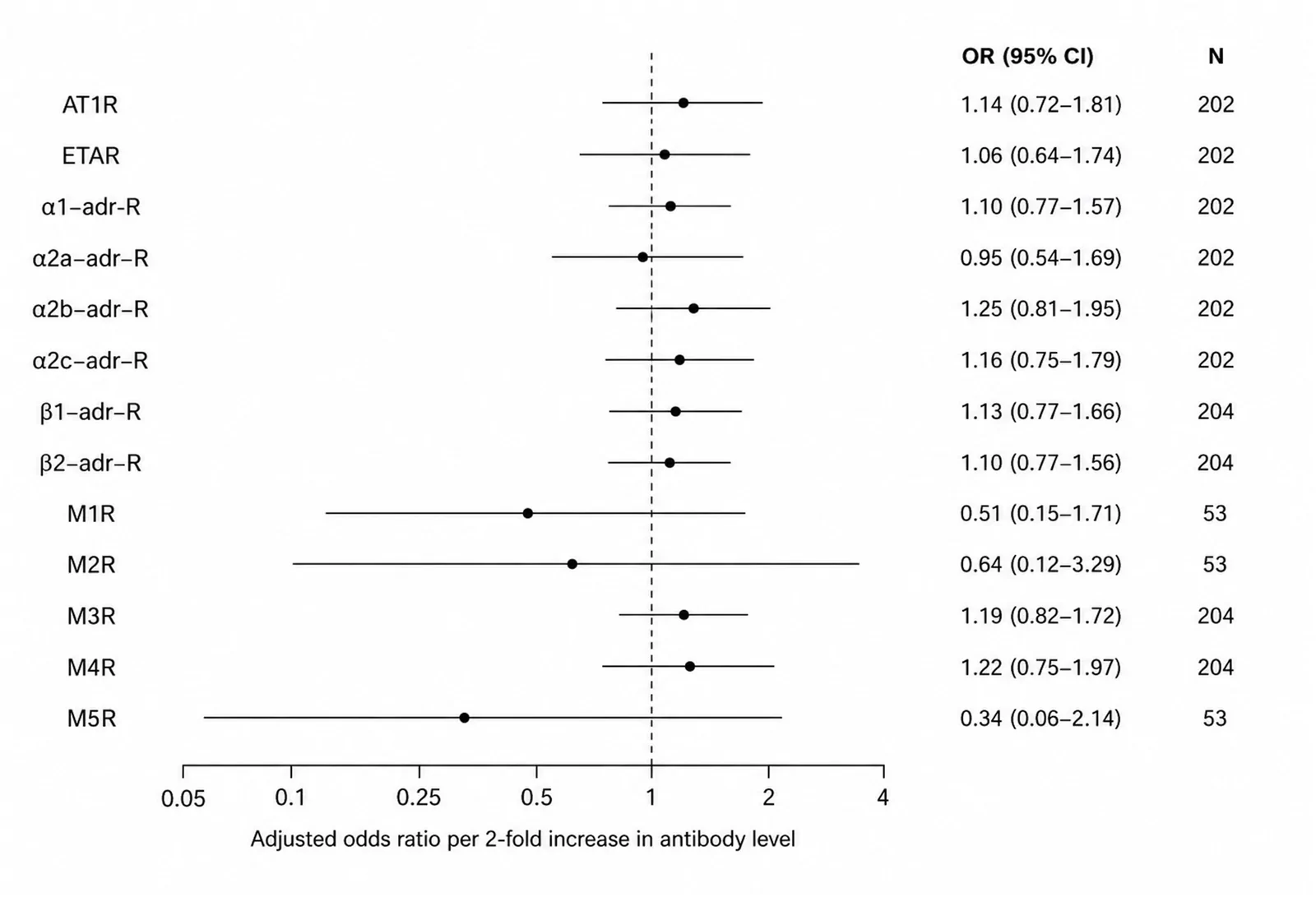
**Association between autoantibody levels and new-onset fatigue.** Forest plot showing adjusted odds ratios (ORs) and 95% confidence intervals for new-onset fatigue associated with a 2-fold increase in circulating autoantibody levels. Estimates were obtained from multivariable logistic regression models adjusted for age, sex, and time since SARS-CoV-2 infection. Time since infection was modeled using a natural cubic spline with three degrees of freedom to account for its nonlinear association with new-onset fatigue. The vertical dashed line indicates an OR of 1.0. ORs >1 indicate higher odds of new-onset fatigue with increasing antibody levels. None of the associations remained statistically significant after false discovery rate (FDR) correction.

### 3.4 Autoantibody positivity across groups

To further assess autoantibody positivity, we determined the proportion of participants in each study group whose autoantibody concentrations exceeded the reference values specified by the manufacturer of the respective ELISA kits (Fig. 3). The proportion of participants exceeding the reference values varied substantially between autoantibodies. The highest proportions were observed for M3R (65.8% in controls, 41.7% in participants with pre-existing fatigue, and 56.2% in participants with new-onset fatigue) and β2-adr-R (55.3%, 45.8%, and 48.6%, respectively). For AT1R, 23.7% of controls, 22.7% of participants with pre-existing fatigue, and 20.1% of participants with new-onset fatigue exceeded the manufacturer-defined reference value. Overall, there was no consistent increase in the proportion of participants exceeding the reference values in the new-onset fatigue group. Fisher's exact tests were performed for each antibody; no significant differences between study groups were observed (all P > 0.05).

**Fig. 3:**
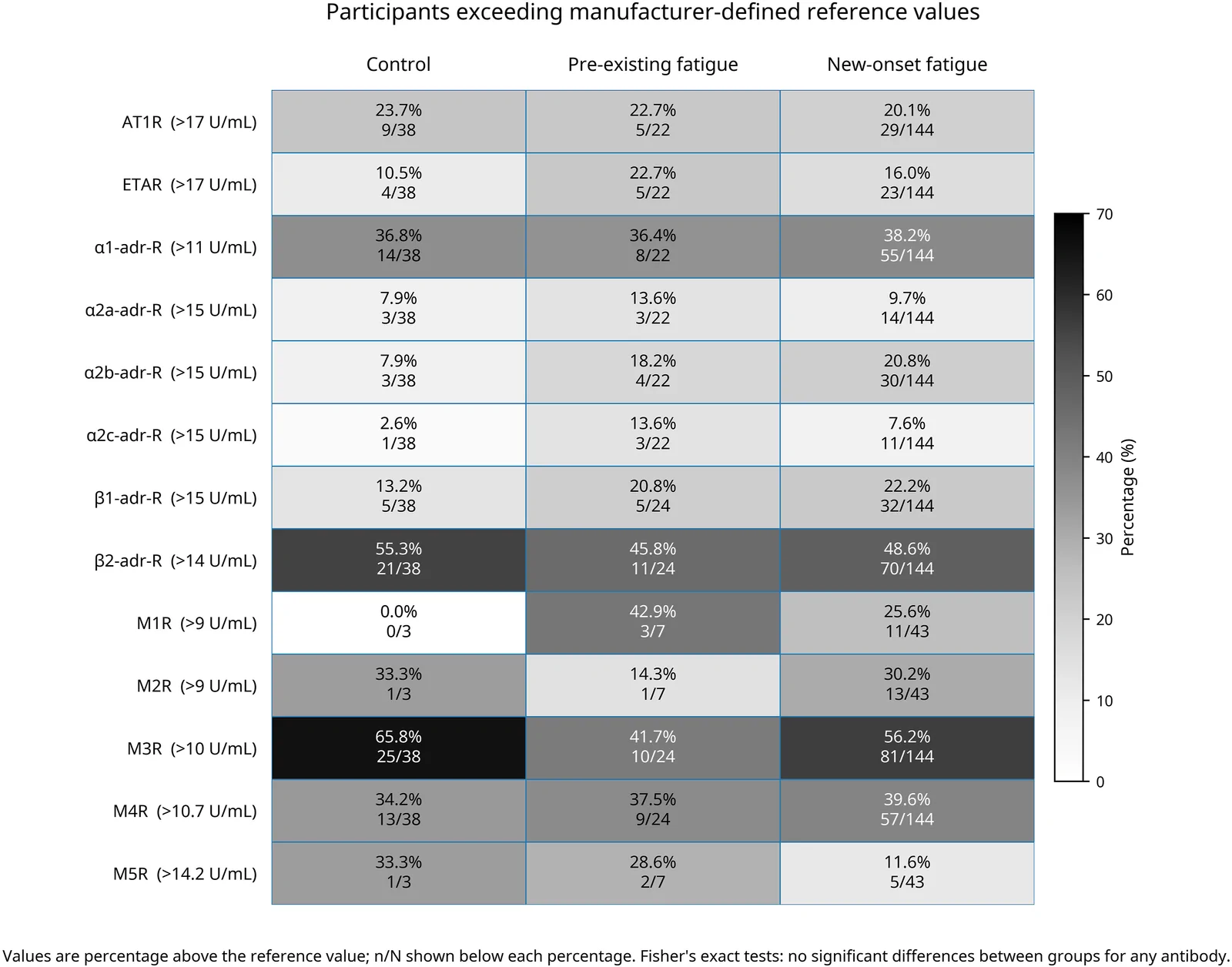
**Proportion of participants with autoantibody levels exceeding manufacturer-defined reference values.** Each row represents one autoantibody, and each column represents the Control, Pre-existing fatigue, and New-onset fatigue groups. Values within the cells indicate the percentage of participants exceeding the respective reference value, with the corresponding number of participants shown as *n/N*. Reference values were defined according to the manufacturers' recommendations for the respective ELISA kits and are indicated next to each antibody. Fisher's exact tests were performed separately for each autoantibody to compare the proportions between the three study groups. No statistically significant differences between groups were observed for any autoantibody (all *P* > 0.05).

As all participants had previously experienced SARS-CoV-2 infection, the present study cannot determine whether SARS-CoV-2 infection itself is associated with increased autoantibody concentrations relative to uninfected individuals.

### 3.5 Cumulative autoantibody positivity and fatigue

To investigate whether, despite the absence of associations for individual autoantibodies, the overall burden of autoantibody positivity was associated with fatigue severity, we calculated the number of autoantibodies exceeding manufacturer-defined reference values for each participant. Based on the eight autoantibodies available for the majority of participants, 97 participants had no positive autoantibodies, 26 had one, 25 had two, and 56 had three or more positive autoantibodies. Fatigue scores did not differ significantly between these categories (Kruskal–Wallis test, χ^2^ = 3.48, df = 3, *P* = 0.323; Table S1). In multivariable linear regression adjusted for age, sex, and time since SARS-CoV-2 infection, with time modeled using a natural cubic spline with three degrees of freedom, cumulative autoantibody positivity was not associated with fatigue score (β = 0.072, 95% CI −0.268 to 0.412, *P* = 0.675; Table S2). Age was positively associated with fatigue score (β = 0.134, 95% CI 0.061–0.208, *P* < 0.001), whereas sex was not (β = −0.367, 95% CI −2.361 to 1.627, *P* = 0.717).

## 4 Discussion

Recent data indicates a significant increase in autoimmune diseases post COVID-19 (Mobasheri et al., 2022; Vahabi et al., 2022; Zebardast et al., 2023), attributed to the emergence of both familiar and previously unidentified autoantibodies (Choutka et al., 2022). Notably, autoantibodies targeting e.g. interferons have been implicated in influencing the severity of COVID-19 (Bastard et al., 2020, 2021) but do not seem to affect long COVID (Achleitner et al., 2024; Peluso et al., 2023). Autoantibodies against neurotransmitter receptors, on the other hand, have been associated with both COVID-19 severity (Cabral-Marques et al., 2022) and long COVID (Camici et al., 2026; Choutka et al., 2022; Wallukat et al., 2021).

In this exploratory study of individuals with previous SARS-CoV-2 infection, we investigated whether binding autoantibodies against 13 neurotransmitter-related receptors were associated with persistent post-COVID fatigue. We found no significant differences in antibody concentrations between participants with new-onset fatigue, pre-existing fatigue, and SARS-CoV-2-infected controls without persistent fatigue. Furthermore, after adjustment for age, sex, and time since SARS-CoV-2 infection, none of the individual autoantibody concentrations was significantly associated with new-onset fatigue. Similarly, no association was observed between individual antibody concentrations and fatigue severity. These findings do not support the use of the measured receptor-binding autoantibodies as stand-alone biomarkers of post-COVID fatigue in this cohort.

In all groups, the percentage of women was higher, consistent with a number of studies showing a greater prevalence of post COVID fatigue among women (Shah et al., 2025). Previously observed sex-based difference in long COVID risk was age, pregnancy, and menopause dependent, with the highest risk among females aged 40 to 55 years (Shah et al., 2025) consistent with our study where the median age of the women experiencing new fatigue (45.5 years) was lower than that of the men (52.4 years).

A substantial proportion of participants exceeded the manufacturer-defined reference values for several of the antibodies examined. Importantly, however, these findings should not be interpreted as evidence that SARS-CoV-2 infection increases receptor-binding autoantibody concentrations. All participants in the present study had previously experienced SARS-CoV-2 infection, and no uninfected control group or pre-infection samples were available. The manufacturer-defined reference values therefore provide information on antibody positivity according to the respective assay but cannot establish an infection-related increase in antibody concentrations. Notably, antibody positivity did not differ significantly between the three study groups, further indicating that exceeding the assay-specific reference values was not associated with the presence of post-COVID fatigue.

Our findings are consistent with the absence of a relationship between receptor-binding autoantibody concentrations and fatigue severity in the present cohort. The inclusion of participants with pre-existing fatigue was particularly informative, as it allowed us to examine whether elevated antibody concentrations were associated with fatigue irrespective of its temporal relationship to SARS-CoV-2 infection. Although some numerical differences between groups were observed for individual antibodies, these differences were not statistically significant, and the multivariable analyses did not identify any antibody for which concentration was independently associated with new-onset fatigue.

We additionally explored whether cumulative antibody positivity might be associated with fatigue severity. Among the eight antibodies available for the majority of participants, neither fatigue scores across categories of cumulative antibody positivity nor multivariable regression analysis showed an association between the number of positive antibodies and fatigue severity. Thus, our data provide no evidence that cumulative positivity for the receptor-binding autoantibodies assessed here is associated with post-COVID fatigue. More complex interactions between individual antibody specificities, however, cannot be excluded by this analysis.

An important consideration is that the assays used in this study measure antibody binding and do not establish functional receptor activity. In particular, they cannot determine whether the detected antibodies activate or inhibit receptor signaling, induce receptor internalization, or otherwise modify receptor function. Therefore, the absence of an association between antibody concentration and fatigue does not establish that functionally active receptor autoantibodies have no biological relevance to post-COVID symptoms. Functional studies will be required to determine whether antibodies identified by binding assays exert biologically meaningful effects and whether such effects differ between clinical phenotypes.

Recent studies have highlighted the multifactorial nature of post-COVID fatigue and ME/CFS, with abnormalities reported across inflammatory and immune pathways, hematopoiesis, lymphocyte and myeloid cell populations, neuronal signaling, and endocrine pathways (Petrov et al., 2026; Shahbaz et al., 2025, 2026). These findings support a broader model in which fatigue may arise from interactions among multiple biological systems rather than from a single circulating factor. Our findings are consistent with this interpretation, as neither individual receptor-binding autoantibody concentrations nor cumulative antibody positivity were associated with fatigue in our cohort. Multidimensional approaches integrating clinical characteristics with immune, metabolic, hematological, neurological, and endocrine biomarkers may therefore be more informative than analyses focused on individual antibody concentrations.

Our study, while insightful, is not without its limitations. First, the study was cross-sectional and all participants had previously experienced SARS-CoV-2 infection. Consequently, we cannot determine whether SARS-CoV-2 infection itself altered receptor-binding autoantibody concentrations or whether the observed antibody positivity represents pre-existing variation. Longitudinal studies including pre- and post-infection samples or appropriately matched uninfected controls will be required to address this question. Secondly, the control group was recruited separately from the fatigue cohort and does not perfectly match the fatigue groups, as the control participants were hospital employees who volunteered for the study, whereas the fatigue groups consisted of patients seeking medical care for post-COVID-19 symptoms. Although age, sex, and time since infection were included as covariates in the multivariable analyses, residual confounding cannot be excluded. Information on vaccination status, reinfection, comorbidities, medications, and hormonal status was not systematically available and could therefore not be incorporated into the models. Third, fatigue was assessed using self-reported questionnaire-based measures rather than specialized objective or multidimensional functional testing. Although such measures may not capture the full complexity of fatigue, they reflect the reality of routine clinical practice, where only a minority of patients undergoes specialized fatigue assessment. Our findings therefore specifically apply to patients assessed using these clinical measures and should not be extrapolated to patients characterized using specialized objective or multidimensional ME/CFS assessments.

Fourth, the pre-existing fatigue group was relatively small, limiting the statistical power to detect differences within this subgroup. Finally, M1R, M2R, and M5R were assessed in substantially fewer participants and should therefore be regarded as exploratory and underpowered analyses.

Despite these challenges, our findings are significant and contribute to the growing understanding of post-COVID fatigue. This exploratory study underscores the importance of considering pre-existing conditions and individual variability in immune responses when investigating post-COVID fatigue. Future research should aim to recruit carefully matched control groups and account for potential confounding factors to further validate these findings and clarify the role of autoantibodies in post-COVID-19 conditions.

It should be noted that currently there are no biomarkers for diagnosing post-COVID fatigue. This makes it difficult to determine which symptoms are truly attributable to SARS-CoV-2 infection and which would have occurred regardless. A recent nationwide population cohort study from Scotland found that 64.5% of individuals reported at least one symptom six months after SARS-CoV-2 infection; however, 50.8% of never-infected individuals also reported symptoms. Therefore, the crude prevalence of one or more symptoms attributable to SARS-CoV-2 infection was 13.8%, 12.8%, and 16.3% at 6, 12, and 18 months, respectively. After adjusting for potential confounders, these figures were even lower (6.6%, 6.5%, and 10.4%, respectively) (Hastie et al., 2023). Therefore, caution is needed when attributing symptoms to prior SARS-CoV-2 infection. These findings may help explain some of the heterogeneity in our study groups and why many patients with fatigue symptoms did not exhibit elevated autoantibodies. They also highlight the urgent need for further research to better understand disease mechanisms, identify biomarkers for diagnostics, and develop effective treatments for post-COVID fatigue. Establishing a clinical score could help correlate individual biomarkers with treatment outcomes (Steenblock et al., 2024). Specifically, multivariable analysis will be key to designing a practical clinical scoring system for monitoring the short- and long-term treatment efficacy of treatments like extracorporeal apheresis in patients with post-COVID fatigue. As noted above, future studies should aim to recruit control groups matched for age, sex, and time since infection to validate and extend our findings.

In conclusion, in this cohort of SARS-CoV-2-infected participants, we found no evidence that concentrations of the measured receptor-binding autoantibodies were associated with new-onset post-COVID fatigue or fatigue severity after accounting for age, sex, and time since infection. Likewise, antibody positivity and cumulative positivity were not associated with fatigue. These findings do not support the use of the measured receptor-binding autoantibodies as stand-alone biomarkers of post-COVID fatigue. They do not, however, address the functional activity of these antibodies or exclude effects in specific clinical subgroups. Further studies combining rigorous clinical phenotyping with longitudinal sampling and functional antibody assays will be required to determine whether specific antibody activities contribute to post-COVID fatigue.

## CRediT authorship contribution statement

**Martin Achleitner:** Writing – review & editing, Project administration, Methodology, Investigation, Conceptualization. **Juliane Dänhardt:** Methodology, Investigation, Data curation. **Romina Kardashi:** Methodology, Data curation. **Nicole Toepfner:** Investigation, Data curation. **Katja de With:** Methodology, Investigation, Data curation. **Zhenfei Li:** Investigation. **Natalia Jarzebska:** Writing – review & editing, Visualization, Validation, Formal analysis. **Roman N. Rodionov:** Writing – review & editing, Supervision, Conceptualization. **Stefan R. Bornstein:** Supervision, Project administration, Funding acquisition, Conceptualization. **Charlotte Steenblock:** Writing – review & editing, Writing – original draft, Visualization, Validation, Supervision, Project administration, Funding acquisition.

## Ethics declaration

Written informed consent to take part in the study and to publish the article has been obtained from all participants or their legal representatives. The privacy rights of participants have been observed.

This study included organ or tissue donors. This study includes human biological material and consent was obtained by donors, or their next of kin or legal representatives, for use in this study and for publication of the article. The samples used in this research were not sourced from executed prisoners or prisoners of conscience.

This study was performed in compliance with relevant laws, regulatory frameworks and guidelines where the research took place. This study was approved by the TU Dresden ethical committee. (Approval No. BO-EK-193052024)

## Data availability

Data is provided within the manuscript.

## Declaration of generative AI and AI-assisted technologies in the manuscript preparation process

During the preparation of this work, the authors used ChatGPT (OpenAI) for language editing and improving the clarity and readability of the manuscript. The author(s) reviewed and edited the output as needed and take full responsibility for the content of the published article.

## Funding

This study was supported by the German Research Foundation (DFG, project no. 314061271 and project no. 288034826 to C.S. and S.R.B).

## Declaration of competing interest

The authors declare that they have no known competing financial interests or personal relationships that could have appeared to influence the work reported in this paper.

## Figures and Tables

**Fig. S1:**
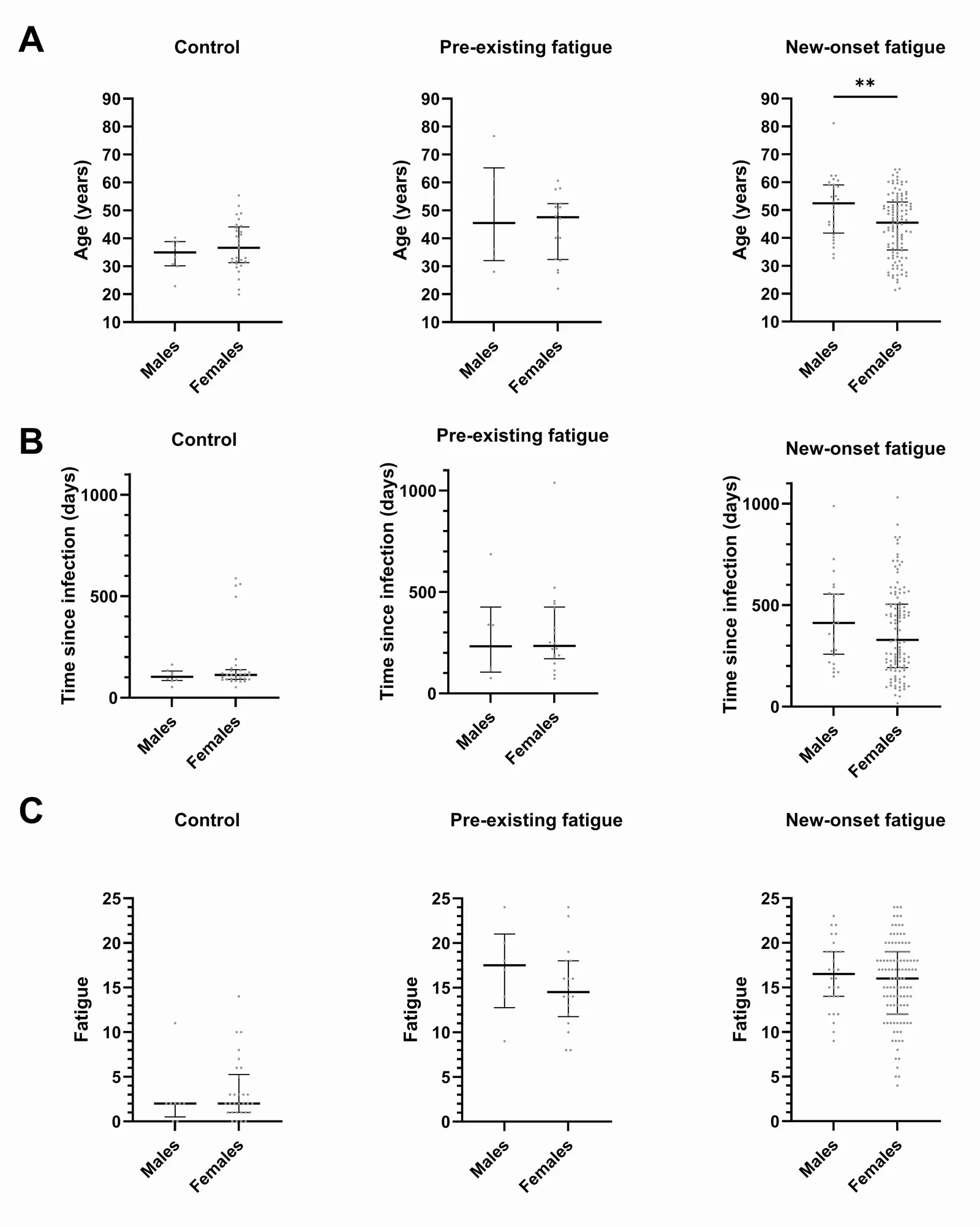
**Age, time since SARS-CoV-2 infection, and fatigue severity according to sex within the study groups.** A) Age according to sex within each study group. B) Time since SARS-CoV-2 infection according to sex within each study group. C) Fatigue scores according to sex within each study group. Individual data points represent individual participants; horizontal lines indicate the median, and error bars indicate the interquartile range. Sex-specific comparisons were performed using the Mann–Whitney *U* test. **p < 0.01.
