# Supplementary material for: Receptor-binding autoantibodies are not associated with post-COVID fatigue
Source: Brain Behav Immun Health. 2026 Oct 23;58:101393. doi: 10.1016/j.bbih.2026.101393 (PMC13720826; doi:10.1016/j.bbih.2026.101393)
Supplement: Supplementary file 1 [file mmc1.docx]

**Table S1. Fatigue scores according to cumulative autoantibody positivity**

| **Number of autoantibodies above reference** | **n** | **Fatigue score, median (IQR)** |
| --- | --- | --- |
| 0 | 97 | 15 (10–18) |
| 1 | 26 | 16 (11.25–18) |
| 2 | 25 | 12 (7–16) |
| ≥3 | 56 | 15 (11–18.25) |
| Total | 204 |  |

Participants were categorized according to the number of eight autoantibodies (AT1R-Ab, ETAR-Ab, α1-adr-R-Ab, α2a-adr-R-Ab, α2b-adr-R-Ab, α2c-adr-R-Ab, β1-adr-R-Ab, and β2-adr-R-Ab) exceeding their respective manufacturer-defined reference values. Fatigue scores are presented as median (IQR). Differences between cumulative autoantibody positivity categories were assessed using the Kruskal–Wallis test. No significant difference in fatigue score was observed between the groups (*P* = 0.323). The analysis included 204 participants with available cumulative autoantibody and fatigue-score data.

**Table S2. Multivariable linear regression analysis of cumulative autoantibody positivity and fatigue score**

| **Variable** | **β** | **95% CI** | **P value** |
| --- | --- | --- | --- |
| **Number of autoantibodies above reference** | 0.072 | -0.268 to 0.412 | 0.675 |
| **Age** | 0.134 | 0.061 to 0.208 | <0.001 |
| **Sex (male vs female)** | −0.367 | −2.361 to 1.627 | 0.717 |

Multivariable linear regression included 202 participants with complete data. The model was adjusted for age, sex, and time since SARS-CoV-2 infection. Time since infection was modeled using a natural cubic spline with three degrees of freedom. β represents the estimated change in fatigue score per one-unit increase in the respective continuous predictor.
